# Two Cases of Eosinophilic Gastroenteritis With Rare Manifestations Revealed in Medical Checkup Findings

**DOI:** 10.7759/cureus.12118

**Published:** 2020-12-17

**Authors:** Tsuyoshi Mishiro, Mamiko Nagase, Makoto Nagasaki, Kyoichi Adachi, Shunji Ishihara

**Affiliations:** 1 Department of Gastroenterology and Hepatology, Faculty of Medicine, Shimane University, Izumo, JPN; 2 Department of Organ Pathology, Shimane University School of Medicine, Izumo, JPN; 3 Department of Pathology, National Hospital Organization Hamada Medical Center, Hamada, JPN; 4 Health Center, Shimane Environment and Health Public Corporation, Matsue, JPN; 5 Department of Gastroenterology, Shimane University Hospital, Izumo, JPN

**Keywords:** eosinophilic gastroenteritis, eosinophilic gastrointestinal disorder, medical checkup, fluoroscopic examination, eosinophil infiltrations

## Abstract

Eosinophilic gastroenteritis (EGE) is characterized by dense infiltration of eosinophils in gastrointestinal tissues, resulting in morphological and functional abnormalities of the gastrointestinal tract. EGE susceptibility is most common among individuals aged 40-50 years old, and hence it is likely that affected patients will be encountered at the time of a medical checkup. In this report, we present two rare cases of EGE that presented interesting manifestations in findings obtained in a fluoroscopic examination performed at an annual medical checkup. Accumulation of case reports is important to provide information to pathologists to enable them to make correct early diagnosis and begin effective treatment at the earliest.

## Introduction

Eosinophilic gastroenteritis (EGE), a type of eosinophilic gastrointestinal disorder (EGID), is a rare chronic inflammatory disease characterized by patchy or diffuse infiltration of eosinophils into different layers of the gastrointestinal tract [1-3]. Affected patients usually have nonspecific abdominal symptoms, such as pain, diarrhea, and vomiting, while many show no specific macroscopic endoscopic abnormality [4,5]. EGE should be diagnosed based on findings obtained in gastrointestinal mucosa histopathological examinations of biopsy specimens [6]. Interestingly, patients with EGE range from those in their teens to the elderly, with a mean age range of 40-50 years [7]; hence, it is likely that affected individuals will be initially encountered at a medical checkup. In this report, we discuss two rare cases of EGE that presented interesting manifestations in the findings of fluoroscopic examinations performed as part of an annual regular medical checkup.

## Case presentation

Case 1

**Figure 1:**
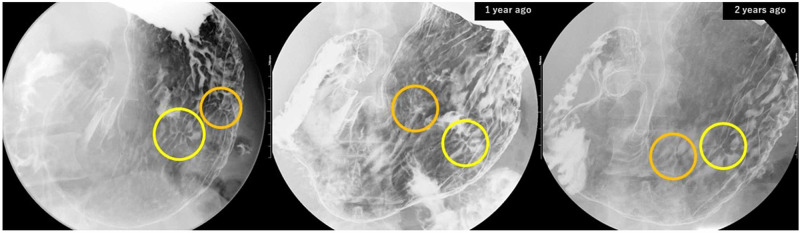
Fluoroscopic imaging performed at a regular annual medical checkup Convergency of gastric folds shown by fluoroscopic imaging performed at a regular annual medical checkup. These abnormalities were observed on the anterior (yellow circle) and posterior (orange circle) walls in the middle-body area of the stomach. Similar traces were also found in images obtained two years prior

A 45-year-old male with epigastric discomfort was found to have an abnormal convergency of gastric folds on X-ray imaging performed as part of a regular medical checkup. The abnormality had been initially detected two years prior, though the patient had not visited a medical facility for a further examination before coming to us. There was no special medical or allergy history, as well as no history of ulcers with subjective symptoms. Blood tests showed only mild glucose intolerance, while tumor markers, antinuclear antibodies, *Helicobacter pylori* (HP) antibodies, and parasite egg tests were all negative. Concentrated folds were observed on the anterior and posterior walls of the stomach in fluoroscopic imaging (Figure 1), with similar traces also found in images obtained two years before at a medical checkup.

**Figure 2:**
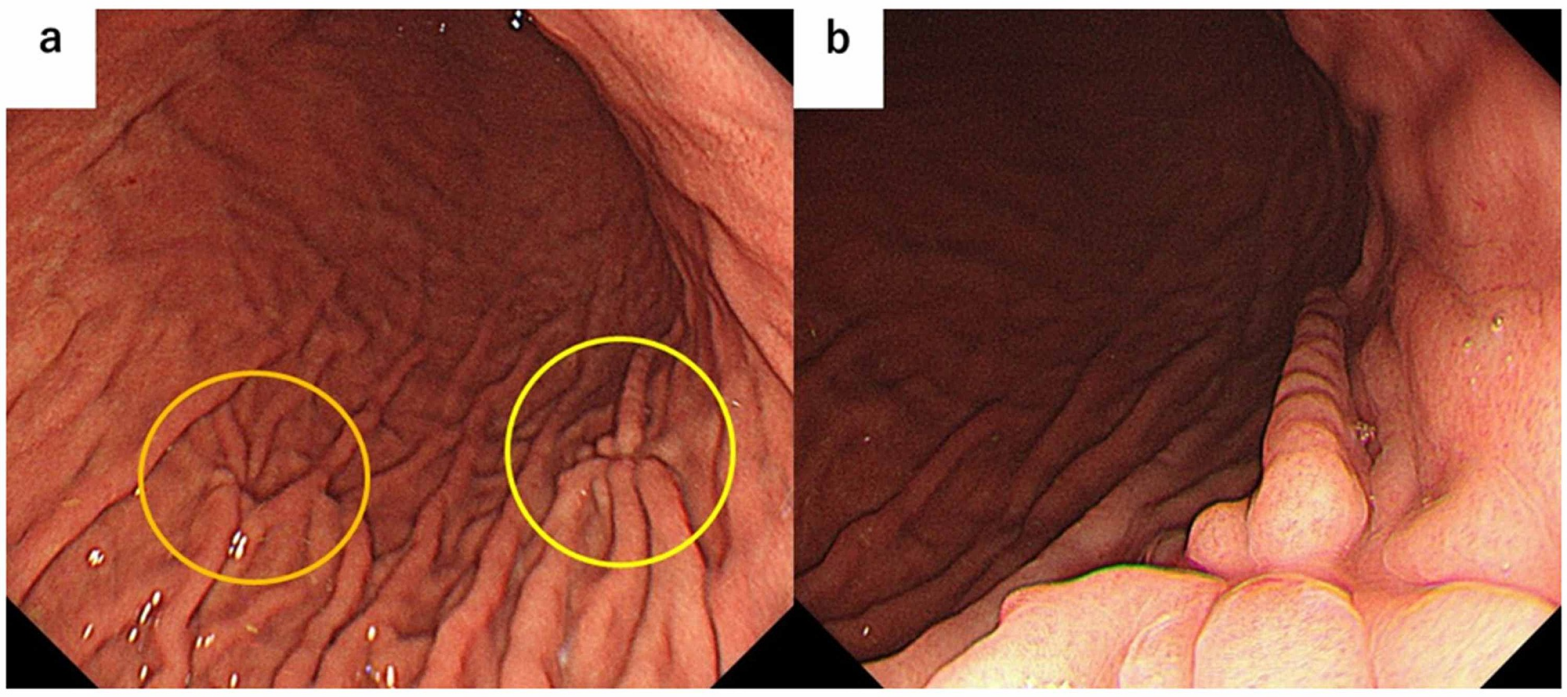
Endoscopic image showing convergency of folds a. Endoscopic image showing convergency of folds on anterior (yellow circle) and posterior (orange circle) walls in the middle-body area of the stomach. b. Endoscopic near-focus image of convergency of folds on the anterior wall, without obvious disruption or fusion

Esophagogastroduodenoscopy (EGD) revealed convergency of folds on the anterior and posterior walls in the middle-body area of the stomach, though there were no obvious fold disruption or fusion findings (Figure 2).

**Figure 3:**
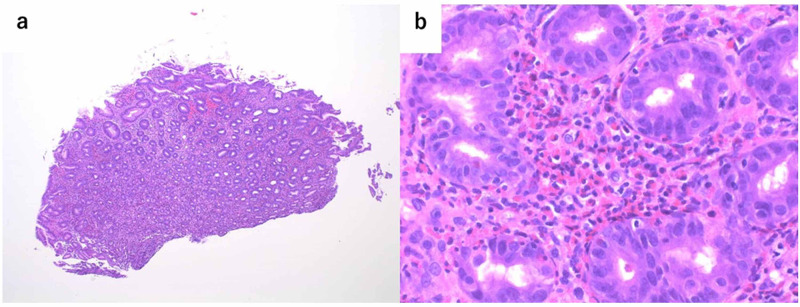
Microscopic image of the gastric ulcer scar site a. Microscopic image of gastric ulcer scar site (hematoxylin-eosin staining, x 40). b. Microscopic image of gastric ulcer scar site (hematoxylin-eosin staining, x 400). Greater than 100 eosinophils infiltrate/HPF were observed in biopsy specimens HPF: high-power field

We obtained biopsy samples from sites close to the center, both yellow and orange ulcer scars. Greater than 100 eosinophil infiltrates per high-power field (HPF) were observed in specimens from the stomach, and also from the mucosa of the duodenum and esophagus (Figure 3).

Since this case was accompanied by eosinophil infiltration through the esophagus, we initially administered a proton pump inhibitor (PPI). Following that administration, the patient immediately showed improved clinical symptoms, and eosinophil infiltration was also dramatically decreased in endoscopic re-examination findings.

Case 2

**Figure 4:**
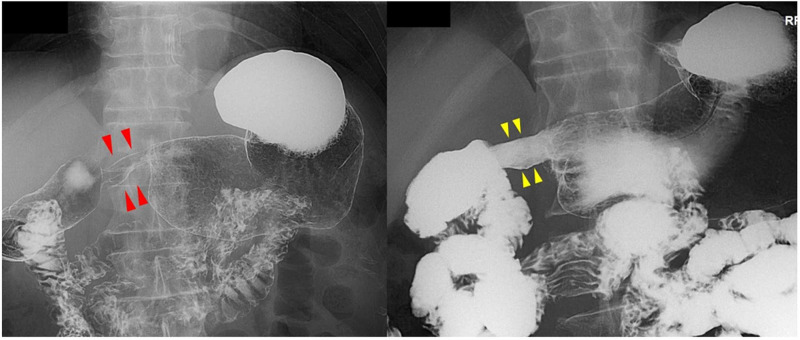
X-ray image showing tunnel-like narrowing of the gastric antral portion X-ray image showing tunnel-like narrowing of the gastric antral portion (left panel, the narrowed portion shown by red arrowhead). The luminal patency of the antral portion was relatively good due to insufflation (right panel, the narrowed portion shown by yellow arrowheads)

A 78-year-old male came to us complaining of postprandial discomfort from food impaction around the epigastric region. Narrowing of the gastric antrum had also been shown on X-ray imaging performed at a regular medical checkup. A local doctor had performed HP eradication treatment two years prior and it had been successful. There was no history of allergy or notable prior drug use. A blood test revealed mild anemia, while immunoglobulin G (IgG) and IgE levels were slightly elevated. White blood cell count was within the normal range and no increase in eosinophil count was observed. Furthermore, antinuclear antibody and parasite egg test results were negative. The antral portion demonstrated a tunnel-like narrowing over approximately 2 cm in X-ray imaging (Figure 4, left panel), though barium passage through the antrum was smooth, and luminal patency in the antral portion was relatively good due to insufflation (Figure 4, right panel).

**Figure 5:**
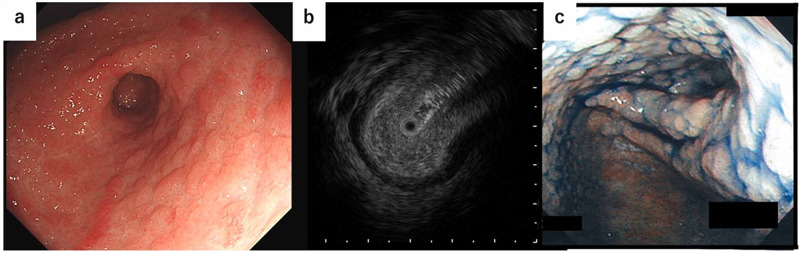
Endoscopic image in the gastric antral portion a. Endoscopic image demonstrating mucosal edema and thickening accompanied with scattered reddish patchy flat elevated lesions in the gastric antral portion. b. Endoscopic ultrasonography showing marked thickening of the antral submucosal layer, though no thickening of the muscular layer or accumulation of peripheral effusion was observed. c. Endoscopic image of the antral portion following indigo carmine spraying obtained at a nearby clinic two years prior. The results indicated a circumferential narrowing of the antral portion, similar to the finding in our examination

EGD revealed that the mucosal surface of the antrum was edematous, thickened, and accompanied with scattered reddish patchy flat elevated lesions, though no erosion areas or ulcers were observed (Figure 5a). The extensibility of the antral portion was relatively good and the scope passed smoothly through the duodenum. Endoscopic ultrasonography (EUS) showed marked thickening of the antral submucosal layer, whereas there was neither thickening of the muscular layer nor accumulation of peripheral effusion (Figure 5b). We later learned that the patient had been examined for EGD at another nearby clinic two years prior, with endoscopic images obtained at that time presented in Figure 5c, which showed a circumferential narrowing of the antral portion, which was similar to our results.

**Figure 6:**
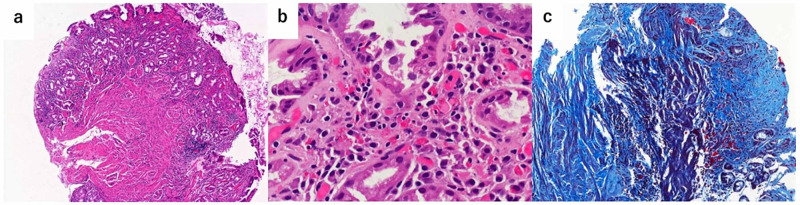
Microscopic image in the gastric antral portion a. Microscopic image showing proliferation of fibrous components under the lamina muscularis mucosae in full-thickness biopsy specimen obtained from the gastric antral portion (hematoxylin-eosin staining, x 40). b. A large number of eosinophil infiltrations (>20/HPF) were observed in the mucosal layer in the overall biopsy specimen obtained from the gastric antral portion (hematoxylin-eosin staining, x 400). c. Masson's Trichrome Staining (x 400) demonstrating the proliferation of collagen fibers in the submucosal layer of a full-thickness biopsy specimen obtained from the narrowed antral portion

Endoscopic mucosal biopsies were obtained from the narrowed portion of the antrum. In pathological findings, a large number of eosinophil infiltrations (>20) per HPF were observed in the mucosal layer (Figure 6b). Additionally, Masson's Trichrome Staining indicated a proliferation of collagen fibers in the submucosal layer, and hence fibrosis and inflammatory cell infiltration were considered to contribute to the antrum narrowing (Figure 6a, Figure 6c).

The patient demonstrated a temporary improvement in symptoms without therapy, though we plan to administer steroids should symptoms recur in the future.

## Discussion

EGID is characterized by dense infiltration of eosinophils in gastrointestinal tissues, resulting in morphological and functional abnormalities of the gastrointestinal tract [1-3]. Although the pathogenesis has yet to be completely elucidated, the disorder is considered to be a type of chronic Th2-type allergic disease caused by food or environmental allergens [8,9].

The most frequently reported symptoms associated with the disease are abdominal pain, diarrhea, and vomiting, though they can vary depending on the site of involvement [7]. Potentially effective therapies recently evaluated include topical glucocorticoid administration, elimination diet, and molecular target therapy with neutralizing antibodies.

EGE is classified into three types based on the extent and layer of the bowel with eosinophil infiltration: mucosal disease, muscular layer disease, and subserosal disease [10]. According to their symptoms, and histological and various imaging findings, including a fluoroscopic examination or EUS, each of the present cases was considered to represent the most frequent type of mucosal disease. Laboratory testing of blood samples, including IgE and Th2-type cytokines, as well as other immune-related substances, are presently not considered reliable for diagnosis or severity grading of EGE [11], and no specific antigen-directed IgE has been detected in EGE patients [12]. Thus, peripheral eosinophilia is thought to be the only good diagnostic clue for EGE, though that may be normal in approximately 20% of affected patients [3]. Both of our patients also had a normal range of concentration of peripheral eosinophils.

Over the past six years, 27 patients have been diagnosed with EGE at our hospital, of whom 13 had large numbers of eosinophilic infiltrates within the stomach confirmed by endoscopic biopsy results. In addition, these 13 patients showed non-specific abnormalities, such as an ulcerative pattern (redness, erosion, ulcer; n=9) or elevated pattern (thickening, nodularity; n=3), or no significant findings (n=1). Therefore, we consider that a histopathological examination of biopsy specimens provides a critical addition to endoscopic study findings, even when no apparent macroscopic endoscopic abnormality is found.

## Conclusions

EGE is an uncommon condition, though affected patients often present with signs and symptoms similar to many other diseases. It is important to share information such as the present case findings to assist pathologists, practitioners, as well as surgeons in making a correct early diagnosis and providing effective treatment to avoid a futile surgical procedure.
